# Recharge and Groundwater Use in the North China Plain for Six Irrigated Crops for an Eleven Year Period

**DOI:** 10.1371/journal.pone.0115269

**Published:** 2015-01-27

**Authors:** Xiaolin Yang, Yuanquan Chen, Steven Pacenka, Wangsheng Gao, Min Zhang, Peng Sui, Tammo S. Steenhuis

**Affiliations:** 1 College of Agronomy and Biotechnology, China Agricultural University, Beijing 100193, People’s Republic of China; 2 Department of Biological and Environmental Engineering, Riley-Robb Hall, Cornell University, Ithaca, NY 14853, United States of America; Potsdam Institute for Climate Impact Research, GERMANY

## Abstract

Water tables are dropping by approximately one meter annually throughout the North China Plain mainly due to water withdrawals for irrigating winter wheat year after year. In order to examine whether the drawdown can be reduced we calculate the net water use for an 11 year field experiment from 2003 to 2013 where six irrigated crops (winter wheat, summer maize, cotton, peanuts, sweet potato, ryegrass) were grown in different crop rotations in the North China Plain. As part of this experiment moisture contents were measured each at 20 cm intervals in the top 1.8 m. Recharge and net water use were calculated based on these moisture measurement. Results showed that winter wheat and ryegrass had the least recharge with an average of 27 mm/year and 39 mm/year, respectively; cotton had the most recharge with an average of 211 mm/year) followed by peanuts with 118 mm/year, sweet potato with 76 mm/year, and summer maize with 44 mm/year. Recharge depended on the amount of irrigation water pumped from the aquifer and was therefore a poor indicator of future groundwater decline. Instead net water use (recharge minus irrigation) was found to be a good indicator for the decline of the water table. The smallest amount of net (ground water) used was cotton with an average of 14 mm/year, followed by peanut with 32 mm/year, summer maize with 71 mm/year, sweet potato with 74 mm/year. Winter wheat and ryegrass had the greatest net water use with the average of 198 mm/year and 111 mm/year, respectively. Our calculations showed that any single crop would use less water than the prevalent winter wheat summer maize rotation. This growing one crop instead of two will reduce the decline of groundwater and in some rain rich years increase the ground water level, but will result in less income for the farmers.

## Introduction

Available water resource has become scarcer especially in arid and semi-arid regions, leading to mining of groundwater in many places [1]. Since agriculture is the largest water user and the increasing and more affluent population consumes more milk and meat products, one of the greatest challenges for the agricultural sector is to produce more food with less water [2]. This is especially critical in the North China Plain (NCP) where agricultural sustainability is threatened by the continued decline in the region’s groundwater table.

The North China Plain is the premier agricultural area in China where 61% of the nation’s wheat, 45% of the maize, 35% of the cotton and 64% of the peanuts are produced [3]. In this area, crop productivity relies heavily on irrigation from groundwater because nearly all usable surface waters are stored in reservoirs for transfer to metropolitan areas [4–6]. Groundwater, and to a limited extent the city waste water downstream of large cities, are used for three to four flood irrigations per year with 300 mm or more water in total [7–10]. Using a large scale weighing lysimeter, Liu et al. [11] reported annual agricultural water consumption for the NCP of about 800 to 900 mm. This is much more than the regional precipitation which averages 480 mm/year (yr) [12]. As a result, crops are irrigated using groundwater as the only available source. The groundwater tables have fallen at a rate of 1m per year in the last 20 years which has led to severe land subsidence in some areas [13, 14] and salt intrusion in areas near the coast.

Earlier regional studies of irrigation efficiency in the North China Plain have used several methods to calculate recharge and evapotranspiration [8, 15–27]. Estimates of actual evapotranspiration (*ET_a_*) of winter wheat varied widely with a low of 346 mm/yr [19] to a high of 737 mm/yr [25] and the *ET_a_* of summer maize from a low of 280 mm/yr [27] to a high 374 mm/yr [22]. Ma et al. [18], Liu et al. [21], Zhang et al. [22], Kong et al. [24] and Zhao et al. [28] used a soil water balance method to calculate *ET_a_* using precipitation and irrigation as input and neglecting deep percolation to below the soil profile. Thus in this method any deep percolation is counted within *ET_a_*. Other researchers accounted for recharge in their simulation models. Sun et al. [8] simulated recharge as a constant fraction of both irrigation and effective rainfall (e.g. Luangcheng County Water Policy and Integrated Water resources Management Office, 1993 [29]) for the period of 1999 to 2002 and found for the NCP a recharge of 25–37 mm under winter wheat in a period including 309–405 mm total irrigation and 347–402 mm precipitation. Ma et al. [27] quantified drainage out of the root zone with a simplification of Darcy’s law, calculating a recharge of winter wheat at 5mm and 64 mm in 2007 (300 mm irrigation and 83 rainfall) and 2008 (210 mm irrigation and 186 rainfall), respectively. Liu et al. [26] calculated percolation from the root zone based on the relation between unsaturated water conductivity and soil moisture content at the bottom of the soil profile, and reported there was 1–18 mm percolation during the winter wheat growing season from 2008 to 2012 with irrigations totaling 226–304 mm, using a root zone was of 1.5 m. However, they did not report the recharge of other crops. Kendy et al. [15, 16] validated a one—dimensional soil—water—balance model assuming that drainage from the soil profile was continuous to estimate areal recharge from winter wheat-summer maize and reported a recharge between 71–170 mm for the whole rotation from 1998 to 2001 under irrigation of 210–328 mm and annual precipitation of 347–402 mm. Using Kendy’s model, Zhang et al. [17] simulated recharge amounts varying between 33–87 mm for winter wheat from 1998 to 2001 with 362–405 mm total irrigation and 53–134 mm precipitation in its growing season. Meanwhile, he also reported that recharge for summer maize was 127 and 12 mm in 2000 (126 mm irrigation and 348 mm precipitation) and 2001 (67 mm irrigation and 212 mm precipitation), respectively. Finally Hu et al. [12] indicated that, based on a combination of SWAT and MODFLOW models, a 39% reduction in irrigation pumping for winter wheat-summer maize would induce groundwater recovery and restoration to the pre-development hydrologic conditions of 1956 in about 74 years in the NCP. As a simplifying assumption, all of the above studies on irrigation efficiency assume either implicitly or explicitly that there is no surface runoff. To neglect surface runoff on the large scale in this area is justified. Since 1980, after the reservoirs were built to collect the river flow from the mountains for industrial and domestic water supply, rivers were dry except in the high rainfall years of 1988 and 1996 [30]. On a small scale, runoff is unlikely due to the high infiltration capacity of the soil and flat topography allowing water to stand in small puddles until it infiltrates or evaporates [12].

In the North China Plain there is no surface water available for irrigation and groundwater is the only source for irrigation. It is useful to know the net ground water use, namely the difference in irrigation water withdrawal and recharge. The excess of irrigation withdrawal above recharge year after year drives the cumulative groundwater level decline. Kendy et al. [15] found that on average, when growing both winter wheat and summer maize, net water use was about 125–212 mm per year from 1998 to 2001. (In this summary, positive values represent greater irrigation than recharge, which is a negative effect on the groundwater level.) Zhang et al. [17] showed net water uses of winter wheat-summer maize from 181–321 mm between 1998 and 2001 in the NCP. Sun et al. [8] reported net water use for winter wheat ranging from 230 mm to 251mm from 1999 to 2002 in the NCP. Sun et al. [31] calculated that the net groundwater use required for winter what-summer maize with reduced input of water and nitrogen under farmers’ practice still surpassed 300 mm yr^-1^ in the NCP. Liu et al. [26] reported that the net water use in the growing season of winter wheat ranged from 226 mm to 292 mm from 2008 to 2012 in the NCP. Based on a root zone model, Ma et al. [27] reported the net water use for winter wheat by itself in the NCP was 146 mm in 2007 and 295 mm in 2008, respectively. However, the models were not directly validated leaving a large uncertainty about recharge and net water use amounts. The exception was the model of Kendy et al. [15, 16] that was validated against observed soil moisture data for the winter wheat-summer maize rotation. They used lysimeter data published by Liu et al. [11] from the Luancheng experimental station site. While these cited reports are very helpful, crop rotation and longer term field research on the net water use and recharge of the staple crops in the NCP remains scare.

Because of limited knowledge about recharge and net water use mentioned above in the NCP, an 11 year experiment was carried out that starting in October 2002 to study the moisture depletion patterns of six crops including winter wheat, summer maize, peanuts, cotton, sweet potato and ryegrass in five cropping systems. In this paper we analyze this experiment during which soil moisture content was measured in increments of 20 cm every 10 days to calculate the net water use of these crops as a function of crop rotation, irrigation schedule and precipitation. The overall objective of this study is to calculate the recharge and the net water use directly based on measured soil moisture content, pan evaporation, irrigation and precipitation for important crops in the North China Plain. Specifically, we will (i) quantify the deep percolation of the six crops from 2003 to 2013; (ii) re-express the difference between deep percolation and irrigation to infer groundwater table change; and (iii) calculate the net water use for the six crops from 2003 to 2013.

## Materials and Methods

### Ethics Statement

This field experiment was conducted in the Luancheng Agro-ecosystem station (37°50’N, 114°40’E, altitude 50 m) which is a long-term experimental site and belongs to the Chinese Academy of Sciences. This research was performed in cooperation with China Agricultural University. The farm operations of this experiment were similar to rural farmers’ operations and did not involve endangered or protected species. This experiment was approved by China Agricultural University and the Chinese Academy of Sciences.

### Study site, soil and climate

**Fig 1:**
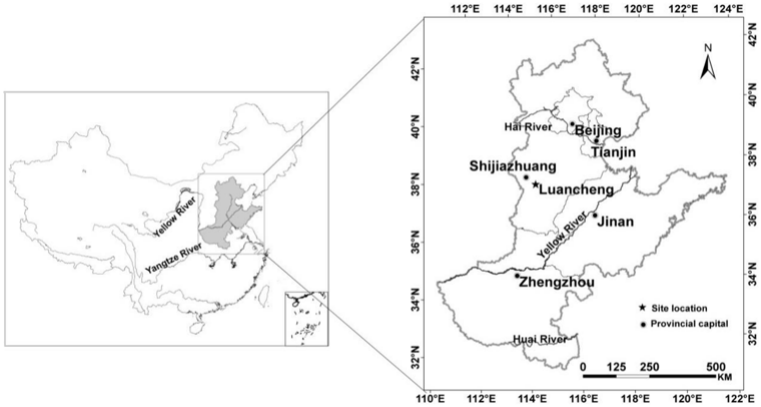
Location of Luancheng County and the North China Plain.

**Fig 2:**
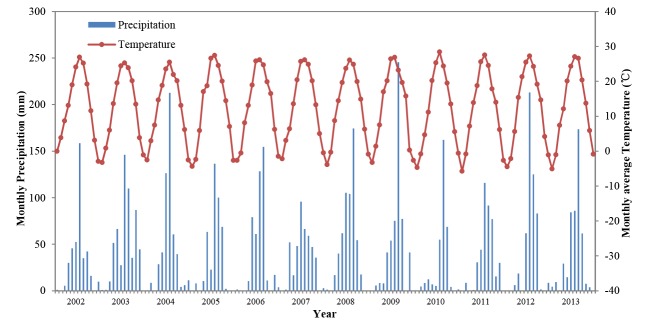
Temporal distribution of monthly precipitation and temperature from 2003 to 2013 at Luancheng experiment site.

The Luancheng Agro-ecosystem station is one of 36 agricultural ecosystem stations in the Chinese Ecological Research Network (CERN). It is located in Luancheng County in Hebei Province. Its property and operation are representative of the agricultural production and climate conditions in the northern part of the North China Plain, where winter wheat-summer maize rotation is the main cropping system (Fig. 1). The water table in Luancheng County has been constantly declining since the 1970s, which fell from 11 m below the surface to about the current 42 m with an annual decline of approximately one m yr^-1^. The experimental site has a warm temperate zone, semi-humid, monsoon climate. The annual mean air temperature is 12°C. The average annual rainfall over the last 20 years was 480 mm, with sharp yearly fluctuations and an erratic seasonal distribution. Generally, 60–70% of the yearly precipitation occurs from June to August. The monthly precipitation and averaged temperature are shown in Fig. 2. The frost-free period is about 200 days from April to October. The experimental site has a sandy loam in the surface layers, light/medium loam at a depth of 40–80 cm, and light clay below 80 cm. The soil profile properties are given in Table 1[32]. The plow layer was about 0.2 m thickness, contained 11 g/kg of organic matter, 1g/kg of total nitrogen, 36 mg/kg of available phosphate, and 96 mg/kg of available potassium. The soil pH is 7.8 [33].

**Table 1:** Soil properties at Luancheng experiment site before the start of experiment.

**Soil depth**	**0–20cm**	**20–40cm**	**40–60cm**	**60–80cm**	**80–100cm**	**100–120cm**	**120–160cm**	**160–200cm**
**Texture**	**Sandy loam**	**Sandy loam**	**Light loam**	**Medium loam**	**Light clay**	**Light clay**	**Light clay**	**Sandy clay**
Bulky density(g/cm^3^)	1.41	1.51	1.47	1.51	1.54	1.64	1.59	1.55
Field capacity(v/v)	0.36	0.35	0.33	0.34	0.34	0.39	0.38	0.38
Wilting point (v/v)	0.096	0.114	0.139	0.139	0.130	0.139	0.164	0.160

### Experimental design and crop management

In October 2002, five cropping systems were established on 15, 4 × 7.5 m plots in a randomized complete-block design with three replicates. Between two plots, there was a 1 m-wide zone without irrigation to minimize cross-plot effect. The five cropping systems were (1) winter wheat—summer maize (WS; 1-year cycle), (2) peanuts→winter wheat—summer maize (PWS; 2-year cycle), (3) rye—cotton→ peanuts→ winter wheat—summer maize (RCPWS; 3-year cycle), (4) sweet potato→ cotton→ sweet potato→ winter wheat—summer maize (SpCSpWS; 4-year cycle), and (5) continuous cotton cropping (Cont C). Planting, harvesting, fertilizers and irrigation applied were the same for each crop independent of its rotation and according to local agronomic practices (Table 2). Fertilizer N (primarily urea fertilizer) was broadcast prior to seedbed preparation and top applications of N, P and K fertilizer were made during the crop growth period. Data were analyzed up to October 2013.

**Table 2:** Experimental treatment of different crops.

	**Winter wheat**	**Summer maize**	**Sweet potato**	**Cotton**	**Peanuts**	**Ryegrass**
Total irrigation amount(mm)	225	105	150	225	150	150
Irrigation time	Sowing/jointing/filling	Jointing/filling	Sowing/middle	Sowing/squaring/boll opening	Sowing/flowering/podding	Sowing/turning green
N(kg/ha)	225	180	70	70	75	225
P_2_O_5_(kg/ha)	112.5	105	97	97	60	112.5
K_2_O(kg/ha)	225	75	128	128	135	225
Planting date	6 Oct	16 Jun	30 Apr	23 Apr	23 Apr	4 Oct
Harvest date	13 Jun	2 Oct	2 Oct	4 Oct	26 Aug	22 Apr

### Data collection

Average daily air temperature, precipitation and pan evaporation were obtained from an automatic weather station located 100m from the experimental plots. The groundwater table was measured about 400 m from the experimental plots at the Experimental Station’s well. Soil volumetric moisture content was measured by a neutron probe (L520) at increments of 20 cm every 10 days to a depth of 180 cm. The top 20 cm soil moisture was measured by a thermo-gravimetric method because neutron probe measurement is inaccurate near the surface.

### Calculations and measurements

Aquifers in the North China Plain are potentially recharged by deep percolation from excess precipitation and irrigation [12, 15, 34]. In order to calculate the cumulative percolation, we calculated the water balance of the top 180 cm for 10 day intervals between consecutive measurements of soil moisture content. Any water shortage in the top 180 cm after subtracting the estimated evapotranspiration by the crop is considered deep percolation which eventually becomes recharge to the groundwater. This assumes implicitly as in other studies within the North China Plain that surface runoff can be neglected [12, 34], which was confirmed by direct observation. It also assumes that interflow is small because the horizontal hydraulic gradient is close to zero in the nearly flat alluvial plain. Finally, with the local groundwater table much deeper than 4 m below the ground surface, capillary rise is negligible [8]. Thus, by neglecting lateral flow and water moving upward from the groundwater, the water balance equation for the deep percolation between moisture measurements for each crop can be written as:

$$DP=\frac{S_{t-\Delta t}-S_{t}+\sum_{t-\Delta t}^{\Delta t}\left(P+I-ET_{a}\right)}{\Delta t}$$ (1)

Where the storage *S* is as measured in the top 180 cm of the soil profile; *S_(t-Δt)_* is the storage in the profile at the previous measurement time; *Δt* is the time between measurements which was usually 10 days; *DP* is the average daily percolation for the interval (mm/day); P is the daily precipitation (mm/day); *I* is the daily irrigation (mm/day); and *ET_a_* is the estimated daily actual evapotranspiration (mm/day). *P*, *I* and *ET_a_* were summed over *Δt* days. *ET_a_* was calculated with the FAO method [35], e.g.

$$ET_{a}=K_{c}P_{c}ET_{p}$$ (2)

Where *K_c_* is the crop growth coefficient; *P_c_* is a pan coefficient that converts the evaporation from a pan to potential evapotranspiration from a well-watered, fully leafed crop; and *ET_p_* is the measured pan evaporation. The crop coefficients *K_c_* varys during the different growth stages of the crop [35]. Here, *P_c_* was equal to 0.7 which was the same as Kendy et al. [15] at the same site. *K_c_* was obtained from the study of Liu et al. [11] that determined values for the Luancheng Station site using lysimeters (S1 Table).

In cases when the deep percolation in Eq. (1), *DP*, was computed to be negative, we assumed that the FAO method (which gives potential evapotranspiration from a very moist surface) overestimated the actual evapotranspiration. Then we reduced the *ET_a_* so that *DP* would become zero in Eq. (1).

Net water use, *NWU*, by the crop from the perspective of the groundwater can be estimated based on *DP* and pumpage for irrigation. The meaning of a negative “use” is that storage in the aquifer has been reduced; a positive “use” means that aquifer storage increased

$$NWU=\sum_{1}^{n}\left(DP-I\right)$$ (3)

Where n was the number of days from sowing till harvest.

### Statistical evaluation

Analysis of variance (ANOVA) was carried out using Statistical Analysis System 9.3 [36] software, and differences were considered significant at the 0.05 level.

## Results and Discussion

### Deep percolation and evaporation

**Fig 3:**
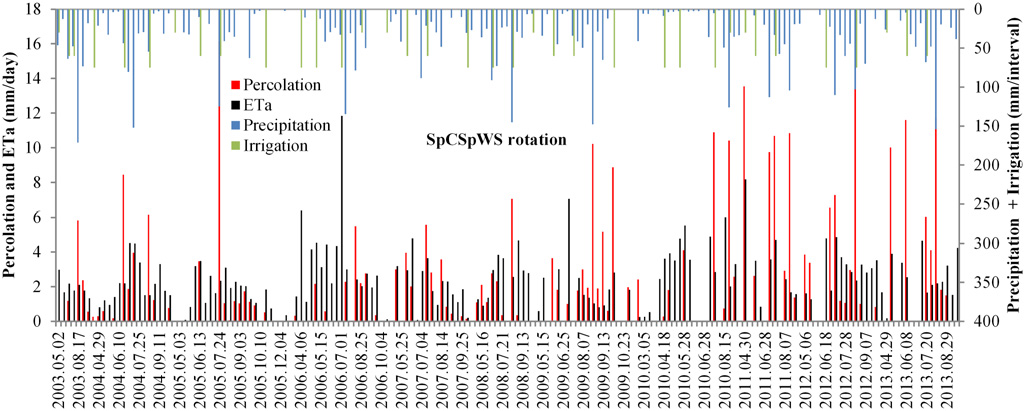
Distribution of daily percolation and *ET_a_* with different precipitation and irrigation in SpCSpWS crop rotation during the period of 2003–2013. (Interval is the days between two measurements; SpCSpWS rotation: sweet potato→ cotton→ sweet potato→ winter wheat—summer maize).

The temporal distributions of precipitation and irrigation and the calculated percolation and evaporation during the period of 2003 to 2013 are presented in Fig. 3 for various crops in the SpCSpWS (sweet potato→ cotton→ sweet potato→ winter wheat—summer maize) rotation. Analogous information for the other four cropping systems is provided in S1 Fig. It is obvious that intensive rains in summer generate more deep percolation than the smaller precipitation events and irrigation applications during the remainder of the year. These findings are consistent with Kendy et al. [15] at the same experimental station and Bradbury et al. [38] in Wisconsin U.S.

By summing the water losses between the soil measurements, the percolation and *ET_a_* for each crop can be calculated with Equations (1) and (2) (Table 3). Results revealed that cotton had significantly more percolation (51–359 mm) than all other crops (P< 0.05), particularly when it was grown as part of the cotton monoculture (Cont C). The reason for the large percolation amounts was the extra irrigation water given to cotton compared to other crops. Liu et al. [39] reported the extra irrigation was needed to meet the water requirement of cotton if there was not much rain. The high standard irrigations, together with the high rainfall in 2004 that fell in a relatively short time period at the beginning of the planting season on relatively wet soil resulted in the greatest percolation of 359 mm under the cotton crop. The minimum percolation for cotton was 51 mm in the following year (2005), mainly because of poor rainfall in that year. Low soil moisture carried over from the preceding cotton in late 2004 also contributed. Irrigation, as mentioned before, was based on a schedule that did not consider soil moisture content.

**Table 3:** Deep percolation of different crops in five crop rotations during the growing season from 2003 to 2013 in the North China Plain (mm).

	**SpCSpWS ^╞^**			**RCPWS ^╞^**			**PWS ^╞^**			**WS ^╞^**			**Cont C ^╞^**		
**Year**	**crops**	**PcP^#^**	**DP^#^**	**crops**	**PcP**	**DP**	**crops**	**PcP**	**DP**	**crops**	**PcP**	**DP**	**crops**	**PcP**	**DP**
2003	SP ^╞^	386	103±4c	R ^╞^	89	154±1a	P ^╞^	338	69±33d	WW	179	129±6b	C	396	161±10a^║^ ^∮^
				C	387	109±4bc				SM	298	72±6d			
2004	C^╞^	502	341±12b	P	468	335±1b	WW	173	0e	WW	129	0e	C	508	359±13a
							SM	398	230±6c	SM	436	100±2d			
2005	SP	392	25±11b	WW	114	66±20a	P	323	60±5a	WW	114	0c	C	392	51±6a
				SM	319	0c				SM	319	0c			
2006	WW^╞^	120	0d	R	15	0d	WW	122	0d	WW	122	0d	C	455	161±15a
	SM^╞^	334	0d	C	435	121±5b	SM	347	6±1d	SM	347	56±3c			
2007	SP	297	0c	P	287	133±5a	P	276	7±4b	WW	140	0c	C	361	138±4a
										SM	296	0c			
2008	C	516	245±1b	WW	222	141±7d	WW	222	63±1e	WW	222	163±3c	C	501	328±4a
				SM	357	0g	SM	348	50±2f	SM	357	0g			
2009	SP	495	136±9b	R	32	0d	P	388	111±13c	WW	119	0d	C	494	246±14a
				C	494	241±4a				SM	407	10±8d			
2010	WW	80	0c	P	230	0c	WW	80	0c	WW	80	0c	C	299	81±7 a
	SM	286	0c				SM	287	0c	SM	287	21±10b			
2011	SP	274	79±6a	WW	73	0e	P	274	13±4d	WW	73	0e	C	360	65±2b
				SM	302	0e				SM	302	31±3c			
2012	C	484	243±2c	R	71	0e	WW	89	0e	WW	79	0e	C	484	294±11b
				C	484	337±11a	SM	475	154±16d	SM	476	159±9d			
2013	SP	421	112±5d	P	359	209±4c	P	359	239±6b	WW	104	0f	C	469	279±9a
										SM	418	42±2e			

^#^ PcP: precipitation in the crop growth period; DP: deep percolation in the crop growth period;

^╞^ SP: sweet potato; C: cotton; WW: winter wheat; SW: summer maize; P: peanuts; R: ryegrass; SpCSpWS: sweet potato→ cotton→ sweet potato→ winter wheat—summer maize rotation; WS: winter wheat—summer maize rotation; PWS: peanuts→winter wheat—summer maize rotation; RCPWS: rye—cotton→ peanuts→ winter wheat—summer maize rotation; Cont C: continuous cotton cropping.

^║^ Different lower case letters after the values in the same row indicate significant difference at the 0.05 level; they do not allow comparing across years (column).

^∮^ +/- indicates mean +/- standard deviation in the replicate groups.

In general, percolation amounts under winter wheat and ryegrass were the least of all crops (in some cases there was no percolation), due to low rainfall (rain stopped generally in the beginning of October) and low initial soil moisture content in the soil profile (Table 3). The long October-May non-precipitation winter wheat cultivation season relied on groundwater irrigation, which was the main reason behind saturated zone storage loss in this area. Some of the irrigation water that we applied would have built up in the soil in winter and spilled downward to the aquifer, but instead was transpired by the winter crops. Similarly to cotton, sweet potato had a relatively high percolation rate ranging from 25 mm to 136 mm. However, there was no percolation in the growing season of sweet potato in 2007, due to the poor precipitation and high evapotranspiration. Peanuts’ growing season percolation ranged from 7 mm to 335 mm (Table 3). Summer maize had the annual average percolation of 44 mm/yr. However, Percolation for all crops was small in 2007 and 2010 because rainfall was scarce (Table 3).

Our results in Table 3 indicate that percolation of water under regionally irrigated cropland is common. This is contrary to the assumptions made by some of the early investigators calculating the crop water use [18, 22, 24, 25]. Therefore, in the next section we demonstrate that our predictions of percolation that recharges the groundwater are realistic and can represent the observed decline in the ground water tables in the North China Plain.

### Validation of deep percolation calculation

**Fig 4:**
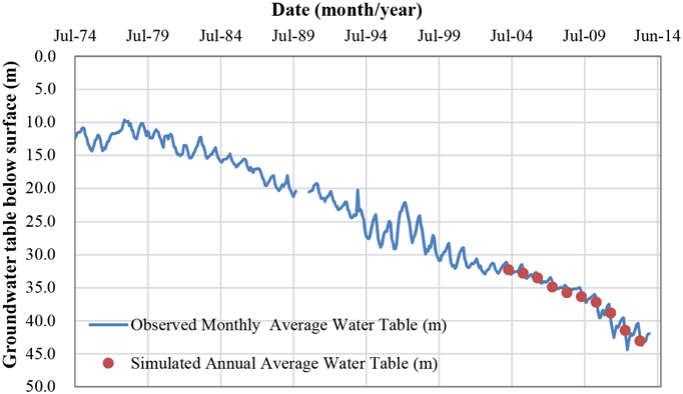
Comparison of the historical observed and simulated groundwater table for winter wheat-summer maize rotation at Luancheng site in the North China Plain.

In order to check the percolation calculation we re-express the difference between the calculated deep percolation and irrigation into infer the groundwater table change. Thus we compare our time series of calculated net water use with the time series of observed groundwater table change at the Luancheng experimental station. Fig. 4 shows the monthly observed water table at the experimental station along with our simulated values which will be explained shortly.

The depth of the groundwater table *d* can be predicted as

$$d_{t}=d_{t-\Delta t}+f\frac{R-I}{\eta }$$ (4)

Where *d* is depth of the ground water in reference to the land surface at zero; R and I are the recharge and irrigation water withdrawn respectively in the period between the current time step, *t*, or the previous time step *t-Δt, f* is the fraction of the land under irrigation and *η* is the specific yield or drainable porosity. *R*, *I* and *d* are in meters.

To calculate the recharge, since the groundwater table below the surface is at 30 m during the experiment period there is a notable time lag between when the water percolates out of the root zone and when it arrives at the groundwater. To estimate this time lag, we look at the ground water table historical behavior. We concentrate on a wet period starting with 1995 with annual rainfall of 510 mm/year; 1996 with 774 mm/year and then 1997 was dry with 272 mm/year. The ground water table as shown in Fig. 4 peaked in 1997 and 1998 indicating that there is one year time delay and the effect is spread over two years.

Since our model is not refined enough to predict the inter-annual variation of the ground water we use a yearly time step. Based on this the average annual recharge $\bar{R}$ is predicted as

$$\bar{R_{y}}=\frac{\bar{DP_{y-1}}+\bar{DP_{y-2}}}{2}$$ (5)

Where the subscript y is the year and $\bar{DP}$ is the annual averaged areal deep percolation.

In order to calculate the annual averaged deep percolation $\bar{DP}$ in the area around the Luancheng experiment station we note that 90% of the area around the Luancheng experiment station is irrigated and 85% is managed under the winter wheat-summer maize rotation [31]. Thus by summing the deep percolation under winter wheat and summer maize (Table 3), we can obtain the annual percolation near the experiment station. In addition based on these observations we assign a value of 0.85 to *f* in Eq. (4).

Since the amount of irrigation is known from table 2 (based on farmer’s practices), the last parameter that need to be estimated is the specific yield in order to find the decline in ground water levels. Specific yield at the site has not been measured. Kendy et al. [15] assumed 0.2 and found a good fit between their computed deep percolation—irrigation and monitor well observations without considering the other factors above. Groundwater modeling literature covering the vicinity assumes 0.10–0.23 [40] and 0.12–0.18 values [12]. Standard texts often cite 0.2 for alluvial gravel and sand like that found beneath the Experiment Station. So we consider an 0.2 value to be reasonable.

The predicted groundwater tables in our winter wheat-summer maize rotation are shown in Fig. 4 after the above calculation. It starts from the year of 2004. Due to this experiment initiated from October 2002, we set a percolation out of the root zone in 2002 the same as in 2003 by lack of a better estimate. As it can be seen in Fig. 4, the predicted annual average groundwater table follow the observed levels well, within the range of monthly variability of the observed water tables. Statistical measures indicate this as well with the root mean square error (RMSE) of 0.6418 m, the mean relative error (RE) of 1.35%, and Nash-Sutcliffe of 0.9633. These three criteria formula to quantify the deviation of the modelling results from the observed data were expressed in Ma et al. [27].

Shu et al., [41] reported among others that the excessive irrigation is generally assumed to be the key factor contributing to declining groundwater tables in the North China Plain. In contrary, especially Eq. (4) distinctly shows that the recharge nor the irrigation by itself are causing the decline of the ground water. It is the difference between the two that is responsible for rate that the ground water changes. Similarly recommendations to decrease the groundwater decline by either decreasing irrigation [12] or increasing the percolation [42] likely should be reconsidered.

### Net Water Use

Exploring groundwater balance Eq. (4), there are two ways to reduce the aquifer depletion by reducing the net water withdrawal (and decreasing the net water use) or by decreasing the fraction of agricultural land (and minimizing the non-evaporation from cropping land). We next explore the choice of crop toward by minimizing the negative balance between irrigation, *I*, and recharge, *R*, using our experiment results.

**Fig 5:**
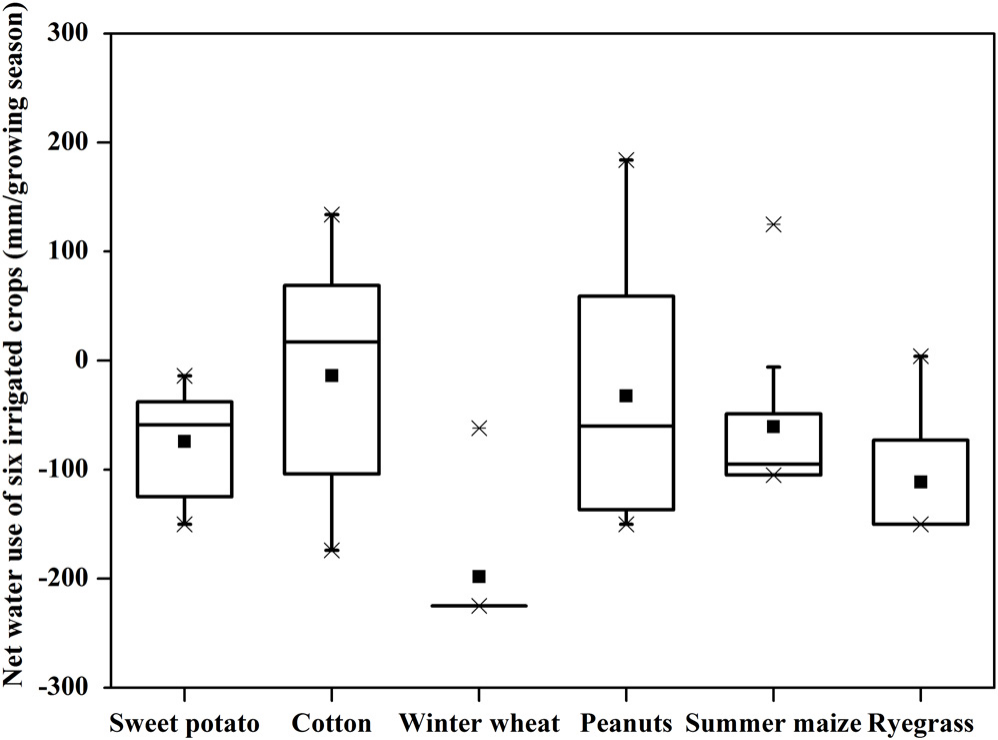
Distribution of Net Water Use (recharge minus irrigation) of six irrigated crops in the growing season from 2003 to 2013 at Luancheng experiment station in the North China Plain. (The box plots show the 25, 50, 75 percentiles. The squares and lines in the box plots indicate the mean and medium, respectively. The crosses indicate the minim und maximum. Negative data indicate the net groundwater depletion.)

Thus to consider what crop is best for decreasing the reduction of groundwater, we calculated the net water use for each crop from the perspective of groundwater using Eq. (3) (Fig. 5). Negative values indicate that irrigation pumpage exceeded recharge, positive that recharge exceeded pumpage. From Fig. 5, the net water withdrawal of winter wheat was greatest among the tested crops with an average of 198 mm/yr in various crop rotations mainly due to the low precipitation in winter and continuous evapotranspiration for regular growth, drawing mostly upon 225 mm irrigation provided in winter wheat growing season. The ryegrass, winter cover crop, with irrigation of 150 mm in each growing season, also had high net water use with an average of 112 mm/yr due to ongoing evapotranspiration and low rainfall in the winter dry season. Under the same 150 mm irrigation, but in a wetter part of the annual weather cycles, sweet potato and peanuts had annual average net water use of 74 mm/yr and 32 mm/yr, respectively. Sweet potato had its highest net water use of 150 mm in 2007 due to the low growing season precipitation and no percolation. Peanuts in the PWS rotation had an extra recharge of 89 mm to the groundwater in 2013. The annual average net water withdrawal of summer maize from the groundwater reached 71mm/yr with the 105 mm irrigation, while in some years summer maize actually replenished the groundwater in the PWS rotation by returning 125 mm in 2004 and 48 mm in 2012. Cotton had the least annual average net water use of 14 mm/yr under its irrigation regime of 225 mm in each growing season. In wet years, cotton was the most likely crop to reverse the water extraction from the groundwater replenishing the aquifer by 18–134mm/yr (S2 Table). Overall, the annual average net water use decreased in the order of winter wheat > ryegrass > sweet potato > summer maize > peanuts > cotton (Fig. 5).

Across all crops, the sharp yearly fluctuations and the erratic seasonal distribution of annual precipitation contributed to a wide variation in any crop’s net water use. Our results are in agreement with Yang et al. [43] who estimated that the crop water requirement for five major crops (wheat, maize, cotton, fruit trees, vegetables) in NCP using crop models DSSAT and COTTON2K, and found winter wheat accounted for over 40% of total irrigation water requirement in the plain, while summer maize and cotton together accounted for 24% of the total irrigation water requirement.

**Fig 6:**
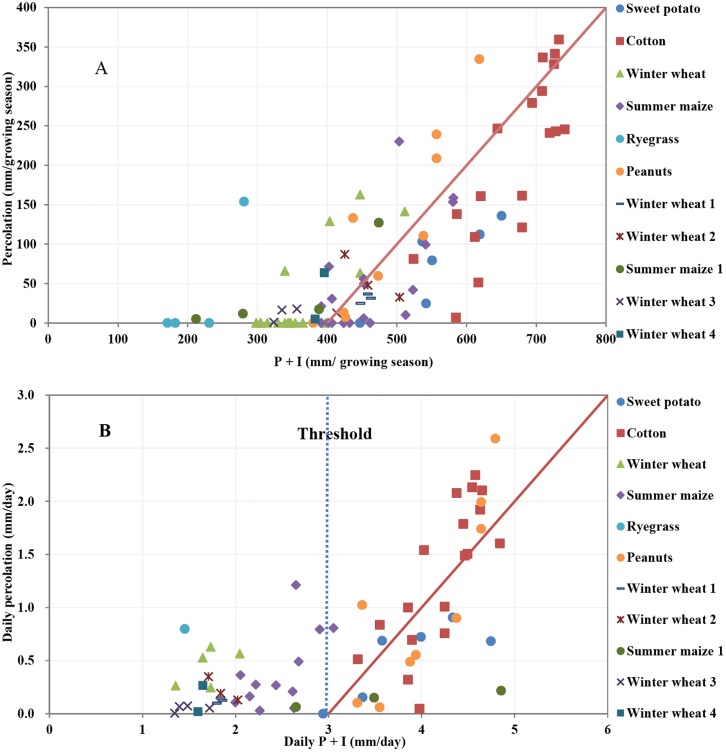
Percolation as a function of precipitation plus irrigation (P+I) during the growing season (A) and daily percolation as a function of the daily (P+I) (B) for different crops in the North China Plain. (In the legend, the data of Sweet potato, Cotton, Winter wheat, Summer maize, Ryegrass and Peanuts come from this study. Winter wheat1 come from Sun et al., (2003) [8]; Winter wheat2 and Summer maize1come from Zhang et al., (2004) [17]; Winter wheat3 come from Liu et al., (2013) [26]; Winter wheat4 come from Ma et al., (2013) [27].)

In order to find the relationship between net water use and rainfall and recharge, Fig. 6A plots the deep percolation (that eventually becomes recharge) versus the sum of precipitation and irrigation (P+I) over the growing season (from sowing to harvest) for each of the crops and for each of the years from 2003 to 2013. Although there is a general relationship in which the percolation increases with increasing P+I, there is a large variation between the various crops. The fit becomes much tighter in Fig. 6B when we take the duration of the growing period into account by dividing the total amounts in the growing season by the number of days of the growing period. Thus in Fig. 6B, we express the percolation versus P+I amounts by expressing the quantities in mm/day. Results reveal that there is a threshold, T, of around 3 mm/day. Above the threshold there is almost a linear relationship between P+I and percolation with a slope close to 1 (Fig. 6B). Below the threshold the percolation varies with the average percolation generally less than 0.75 mm/day with an average of approximately 0.2 mm/day.

The equation for the line for T≥3 mm/day in Fig. 6B can be written as

$$\bar{DP}\approx \bar{P}+\bar{I}-T$$ (6)

Nothing that the threshold value is approximately equal to the average evapotranspiration during the growing season we can rewrite Eq. (6) as

$$\bar{DP}\approx \bar{P}+\bar{I}-\bar{ET}\;\text{for}\;\bar{P}+\bar{I}\geq \bar{ET}$$ (7)

The simple results for estimating the amount of deep percolation per crop in Eq. (7) is based on regression of experimental results of percolation amounts determined from frequently measured moisture contents during the growing season and is therefore different from Eq. (1) that is based on theoretical considerations.

To show that this Eq. (7) is valid for other places as well in the North China plain we plotted in Fig. 6A and 6B the result of these other studies. Zhang et al. [17] reported that the recharge of winter wheat ranged from 33–87 mm from 1998 to 2001 with the total P+I from 425 to 504 mm in its growing season and the recharge of summer maize ranged from 5–127 mm with the total P+I from 212–474 mm. Sun et al. [8] found that the recharge of winter wheat ranged from 25–37mm from 1999 to 2002 under the total P+I of 448–463 mm during the growing season. Liu et al. [26] demonstrated that the recharge of winter wheat ranged from 1–18 mm from 2009 to 2012 with the total P+I of 323–357 mm. Ma et al. [27] reported that the recharge of winter wheat was from 5 mm to 64 mm with water input (P+I) of 383 to 396 mm during the growing season from 2007 to 2009. These studies provide similar estimations of evapotranspiration and net water use for winter wheat and summer maize to our study in the same region. Our study, however, also consider the net water use by cotton, sweet potato, peanuts, ryegrass.

Rewriting Eq. (7) and combine it with Eq. (4) we find that the change in water table can be written as

$$d_{y}=d_{y-1}+f\frac{P_{y-1}+P_{y-2}-ET_{y-1}-ET_{y-2}}{2\eta }$$ (8)

It clearly shows that the *ET* term is the most important one in managing groundwater. Thus the only way to maintain yield and lessen the impact on groundwater is by reducing non-productive evapotranspiration from crops in the area’s agriculture. This is actively being pursued, such as breeding new drought resistant crop [44], increasing soil surface management (tillage [45]); soil nutrient management [46]; crop residue cover and mulching [47]; irrigation management [48, 49]; Meanwhile, new alternative cropping systems were also recommended to develop [31, 50].

In this study, the establishment of the relationship between recharge and precipitation plus irrigation and the estimation of the groundwater decline change provide a practical theoretical basis for improving irrigation regimes, reducing unproductive evaporation, and ultimately more effective utilization of the limited water resources in this area.

## Conclusions

In the North China Plain, irrigation is often essential to achieve economically viable crop production. More accurate insights about seasonal aquifer recharge and net water use are prerequisites to calculating the groundwater balances at local, sub-watershed scales for effective management of scare water resources in this area. In this study, the recharge and net water use of six crops were quantified from 2003 to 2013 based on a long-term crop rotation field experiment using simple soil water balance monitoring. Results showed that the annual average net water use (recharge minus irrigation) was highest beneath winter wheat averaging 198 mm/yr, followed by ryegrass with 112 mm/yr, sweet potato with 74 mm/yr, summer maize with 71mm/year, peanuts with 32 mm/yr, and cotton with the lowest at 14 mm/yr. Moreover, the groundwater table prediction and the establishment of the relationship between percolation and precipitation plus irrigation provide an important perspective when searching for efficient irrigation regimes and sustainable water management policy. This study implicated that groundwater decline will be less by growing different crops that require less time to mature, evaporate less than the potential rate. In some years with above average rainfall this might even lead to an increase in groundwater tables.

## Supporting Information

S1 Fig Distributions of daily percolation and *ET_a_* with different precipitation and irrigation in various crop rotations during the period of 2003–2013. (A) WS: winter wheat—summer maize rotation. (B) PWS: peanuts→winter wheat—summer maize rotation. (C) RCPWS: rye—cotton→ peanuts→ winter wheat—summer maize rotation. (D) Cont C: continuous cotton.
(TIF)

S1 Table The crop coefficient (Kc) values of different crops at Luancheng experiment site. (DOC)

S2 Table Net water use (recharge minus irrigation) of each crop in each crop rotation of each year’s growing season from 2003 to 2013 at Luancheng site in the North China Plain (mm). (DOC)

## References

[1] Yang JF, Wan SQ, Deng W, Zhang GX (2007) Water fluxes at a fluctuating groundwater and groundwater contributions to wheat water use in the lower Yellow River flood plain, China. Hydrol Process 21: 71–724. 10.1002/hyp.6246

[2] Zwart S, Bastiaanssen W (2004) Review of measured crop water productivity values for irrigated wheat, rice, cotton and maize. Agric Water Manage 69 (2): 115–133. 10.1016/j.agwat.2004.04.007

[3] National Bureau of Statistics of China (2008) China Statistical Yearbook 2008. China Statistics Press, Beijing.

[4] Fang QX, Ma L, Green TR, Yu Q, Wang TD, et al (2010) Water resources and water use efficiency in the North China Plain: Current status and agronomic management options. Agric Water Manage 97 (8): 1102–1116. 10.1016/j.agwat.2010.01.008

[5] Mo XG, Liu SX, Lin ZH, Guo RP (2009) Regional crop yield, water consumption and water use efficiency and their responses to climate change in the North China Plain. Agric Ecosyst Environ 134: 67–78. 10.1016/j.agee.2009.05.017

[6] Moiwo JP, Yang Y, Li H, Han S, Yang Y (2010) Impact of water resource exploitation on the hydrology and water storage in Baiyangdian Lake. Hydrol Process 24: 3026–3039. 10.1002/hyp.7716

[7] Zhang Z, Cui Y, Chen Z, Xu P, Huang L, et al (2003) Discussion of water resource balance and water saving agriculture development in Huabei Plain. Review of China Agriculture and Technology 5 (4): 42–47. [in Chinese with English Abstract].

[8] Sun HY, Liu CM, Zhang XY, Shen YJ, Zhang YQ (2006) Effects of irrigation on water balance, yield and WUE of winter wheat in the North China Plain. Agric Water Manage 85 (1–2): 211–218. 10.1016/j.agwat.2006.04.008

[9] Sun HY, Shen YJ, Yu Q, Flerchinger GN, Zhang YQ, et al (2010) Effect of precipitation change on water balance and WUE of the winter wheat-summer maize rotation in the North China Plain. Agric Water Manage 97 (8): 1139–1145. 10.1016/j.agwat.2009.06.004

[10] Zhao RF, Chen XP, Zhang FS, Zhang HL, Schroder J, et al (2006) Fertilization and nitrogen balance in a wheat-maize rotation system in North China. Agron J 98 (4): 938–945. 10.2134/agronj2005.0157

[11] Liu CM, Zhang XY, Zhang YQ (2002) Determination of daily evaporation and evapotranspiration of winter wheat and maize by large-scale weighing lysimeter and micro-lysimeter. Agric Forest Meteorol 111 (2): 109–120. 10.1016/S0168-1923(02)00015-1

[12] Hu YK, Moiwo JP, Yang YH, Han SM, Yang YM (2010) Agricultural water-saving and sustainable groundwater management in Shijiazhuang Irrigation District, North China Plain. J Hydrol 393: 219–232. 10.1016/j.jhydrol.2010.08.017

[13] Jia JS, Liu CM (2002) Groundwater dynamic drift and response to different exploitation in the North China Plain: a case study of Luancheng County, Hebei Province. Acta Geographica Sinica 57 (2): 201–209.

[14] Chen JY, Tang CY, Shen YJ, Sakura Y, Kondoh A, et al (2003) Use of water balance calculation and tritium to examine the dropdown of groundwater table in the piedmont of the North China Plain (NCP). Environ Geol 44 (5): 564–571. 10.1007/s00254-003-0792-3

[15] Kendy E, Gerard-Marchant P, Walter MT, Zhang YQ, Liu CM, et al (2003) A soil-water balance approach to quantify groundwater recharge from irrigated cropland in the North China Plain. Hydrol Process 17: 2011–2031. 10.1002/hyp.1240

[16] Kendy E, Zhang YQ, Liu CM, Wang JX, Steenhuis T (2004) Groundwater recharge from irrigated cropland in the North China Plain: case study of Luancheng County, Hebei Province, 1949–2000. Hydrol Process 18 (12): 2289–2302. 10.1002/hyp.5529

[17] Zhang YQ, Kendy E, Yu Q, Liu CM, Shen YJ, et al (2004) Effect of soil water deficit on evapotranspiration, crop yield, and water use efficiency in the North China Plain. Agric Water Manage 64 (2): 107–122. 10.1016/S0378-3774(03)00201-4

[18] Ma L, Sui P, Gao WS, Li FR (2008) Water resources use efficiency of different cropping patterns in the Piedmont of Mt.Taihang. Agricultural research in the arid areas 26 (2): 177–183. [in Chinese with English Abstract].

[19] Zhang XY, Chen SY, Sun HY, Dong P, Wang YM (2008) Dry matter, harvest index, grain yield and water use efficiency as affected by water supply in winter wheat. Irrig Sci 2: 1–10. 10.1007/s00271-008-0131-2

[20] Zhang XY, Wang YZ, Sun HY, Chen SY, Shao LW (2013) Optimizing the yield of winter wheat by regulating water consumption during vegetative and reproductive stages under limited water supply. Irrig Sci 31: 1103–1112. 10.1007/s00271-012-0391-8

[21] Liu HJ, Yu LP, Luo Y, Wang XP, Huang GH (2011) Responses of winter wheat (Triticum aestivum L.) evapotranspiration and yield to sprinkler irrigation regimes. Agric Water Manage 98 (4): 483–492. 10.1016/j.agwat.2010.09.006

[22] Zhang M, Sui P, Chen YQ, Sun ZG, Ma L (2011) Water consumption characteristics of alternative crop rotation in the piedmont of Mt. Taihang. Chinese Agricultural Science Bulletin 27 (20): 251–257. [in Chinese with English Abstract].

[23] Huo ZL, Feng SY, Huang GH, Zheng YY, Wang YH, et al (2012) Effect of groundwater level depth and irrigation amount on water fluxes at the groundwater table and water use of wheat. Irrig Drain 61: 348–356. 10.1002/ird.685

[24] Kong FL, Cai WT, Shi LG, Chen F (2012) The characteristics of annual water consumption for winter wheat and summer maize in North China Plain. Procedia Engineering 28: 376–381. 10.1016/j.proeng.2012.01.736

[25] Fan ZL, Chai Q, Huang GB, Yu AZ, Huang P, et al (2013) Yield and water consumption characteristics of wheat/maize intercropping with reduced tillage in an Oasis region. Eur J Agron 45: 52–58. 10.1016/j.eja.2012.10.010

[26] Liu XW, Shao LW, Sun HY, Chen SH, Zhang XY (2013b) Responses of yield and water use efficiency to irrigation amount decided by pan evaporation for winter wheat. Agric Water Manage 129: 173–180. 10.1016/j.agwat.2013.08.002

[27] Ma Y, Feng SY, Song XF (2013) A root zone model for estimating soil water balance and crop responses to deficit irrigation in the North China Plain. Agric Water Manage 127, 13–24. 10.1016/j.agwat.2013.05.011

[28] Zhao LY, Liu YL, Pan ZH, An LP, Pan XB, et al (2013) Impact of recent climate change on dry-land crop water consumption in the Northern Aro-Pastoral Transitional Zone of China. Acta Meteor Sinica 27(4): 585–590. 10.1007/s13351-013-0405-3

[29] Luancheng County Water Policy and Integrated Water Resources Management Office (1993) Investigation Report on Current Development and Use of Water Resources (Shuiziyuan Kaifa Liyong Xianzhuang Diaocha Baogao). Shijiazhuang City, Luancheng County, Hebei Province.

[30] Yang Y, Watanabe M, Sakura Y, Changyuan T, Hayashi S. (2002) Groundwater-table and recharge changes in the Piedmont region of Taihang Mountain in Gaocheng City and its relation to agricultural water use. Water SA. 28: 171–178. 10.4314/wsa.v28i2.4883

[31] Sun QP, Kröbel R, Müller T, Römheld V, Cui ZL et al (2011) Optimization of yield and water-use of different cropping systems for sustainable groundwater use in the North China Plain. Agric Water Manage 98: 808–814. 10.1016/j.agwat.2010.12.007

[32] Chen C, Wang EL, Yu Q (2010) Modelling the effects of climate variability and water management on crop water productivity and water balance in the North China Plain. Agric Water Manage 97: 1175–1184. 10.1016/j.agwat.2008.11.012

[33] Zhang MY, Wang FJ, Chen F, Malemela MP, Zhang HL (2013) Comparison of three tillage systems in the wheat-maize system on carbon sequestration in the North China Plain. J Clean Prod 54: 101–107. 10.1016/j.jclepro.2013.04.033

[34] Yang YH, Watanabe M, Zhang XY, Zhang JQ, Wang QX, et al (2006) Optimizing irrigation management for wheat to reduce groundwater depletion in the piedmont region of the Taihang Mountains in the North China Plain. Agric Water Manage 82: 25–44. 10.1016/j.agwat.2005.07.020

[35] Allen RG, Pruitt WO, Wright JL, Howell TH, Ventura F, et al (2006) A recommendation on standardized surface resistance for hourly calculation of reference ET_o_ by the FAO 56 Penman-Monteith method. Agric Water Manag 81: 1–22. 10.1016/j.agwat.2005.03.007

[36] Sas I.I. (2011) SAS/STAT user’s guide, version 9.3 SAS Institute, Cary, NC, USA.

[37] Yang XL, Sui P, Chen YQ, Gao WS (2014) Data from: Recharge and groundwater use in the North China Plain for six irrigated crops for an eleven year period. http://hdl.handle.net/1813/37320. 10.1371/journal.pone.0115269 PMC4308074 25625765

[38] Bradbury KR, Dripps WR, Hankley C, Anderson MP, Potter KW (2000) Refinement of two methods for estimation of groundwater recharge rates. Final Project Report, Wisconsin Department of Natural Resources, Madison, Wisconsin.

[39] Liu MX, Yang JS, Li XM, Yu M, Wang J (2013) Numerical simulation of soil water dynamics in a drip irrigated cotton field under plastic mulch. Pedosphere 23 (5): 620–635. 10.1016/S1002-0160(13)60055-7

[40] Lin XY, Yang YS (1991) The Optimization of Groundwater Supply System in Shi Jiazhuang City, China. Wat. Sci. Tech, 24(11):71–76.

[41] Shu YQ, Villholth KG, Jensen KH, Stisen S, Lei YP (2012) Integrated hydrological modeling of the North China Plain: options for sustainable groundwater use in the alluvial plain of Mt. Taihang. J Hydrol 464–465: 79–93. 10.1016/j.jhydrol.2012.06.048

[42] Currell MJ, Han DM, Chen ZY, Cartwright I (2012) Sustainability of groundwater usage in northern China: dependence on palaeowaters and effects on water quality, quantity and ecosystem health. Hydrol. Process. 26, 4050–4066. 10.1002/hyp.9208

[43] Yang YM, Yang YH, Moiwo JP, Hu YK (2010) Estimation of irrigation requirement for sustainable water resources reallocation in North China. Agric Water Manage 97: 1711–1721. 10.1016/j.agwat.2010.06.002

[44] Passioura J (2006) Increasing crop productivity when water is scarce-from breeding to field management. Agric Water Manage 80: 176–196. 10.1016/j.agwat.2005.07.012

[45] Hou XQ, Li R, Jia ZK, Han QF, Wang W, et al (2012) Effect of rotational tillage practices on soil properties, winter wheat yields and water-use efficiency in semi-arid areas of north-west China. Field Crop Res 129: 7–13. 10.1016/j.fcr.2011.12.021

[46] Fang QX, Yu Q, Wang EL, Chen YH, Zhang GL, et al (2006) Soil nitrate accumulation, leaching and crop nitrogen use as influenced by fertilization and irrigation in an intensive wheat-maize double cropping system in the North China Plain. Plant and Soil 284: 335–350. 10.1007/s11104-006-0055-7

[47] Qin W, Chi BL, Oenema O (2013) Long-term monitoring of rainfed wheat yield and soil water at the loess plateau reveals low water use efficiency. Plos One. 8 (11): e78828 10.1371/journal.pone.0078828 24302987 PMC3841156

[48] Cui NB, Du TS, Kang SZ, Li FS, Zhang JH, et al (2008) Regulated deficit irrigation improved fruit quality and water use efficiency of pear-jujube trees. Agric Water Manage 95: 489–497. 10.1016/j.agwat.2007.11.007

[49] Hu TT, Kang SZ, Li FS, Zhang JH (2009) Effects of partial root-zone irrigation on the nitrogen absorption and utilization of maize. Agric Water Manage 96: 208–214. 10.1016/j.agwat.2008.07.011

[50] Ye YL, Xiao YB, Huang YF, Li L (2008) Effect of wheat/maize and faba bean/ maize intercropping on water use. Chinese Agricultural Science Bulletin 24: 445–449.

