# Supplementary figures and images for: Recharge and Groundwater Use in the North China Plain for Six Irrigated Crops for an Eleven Year Period
Source: PLoS One. 2015 Jan 27;10(1):e0115269. doi: 10.1371/journal.pone.0115269 (PMC4308074; doi:10.1371/journal.pone.0115269)

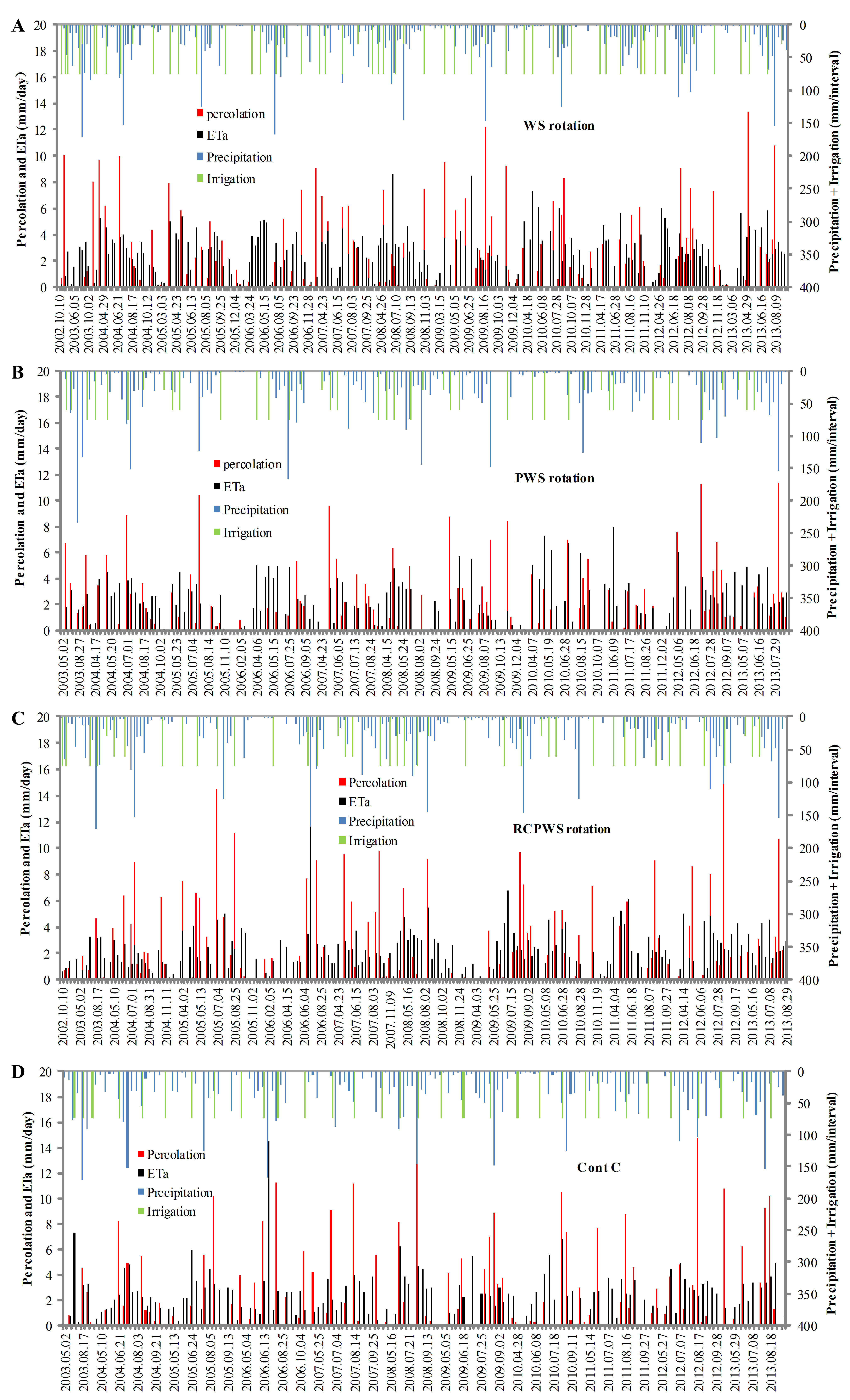

Supplement: S1 Fig — (A) WS: winter wheat—summer maize rotation. (B) PWS: peanuts→winter wheat—summer maize rotation. (C) RCPWS: rye—cotton→ peanuts→ winter wheat—summer maize rotation. (D) Cont C: continuous cotton. (TIF) [file pone.0115269.s001.tif]
