# Supplementary material for: Recharge and Groundwater Use in the North China Plain for Six Irrigated Crops for an Eleven Year Period
Source: PLoS One. 2015 Jan 27;10(1):e0115269. doi: 10.1371/journal.pone.0115269 (PMC4308074; doi:10.1371/journal.pone.0115269)
Supplement: S1 Table — (DOC) [file pone.0115269.s002.doc]

**Supporting Information**

**Table S1 The crop coefficient (Kc) values of different crops in Luancheng experiment site.**

| Kc/ month | Jan | Feb | Mar | Apr | May | Jun | Jul | Aug | Sep | Oct | Nov | Dec |
| --- | --- | --- | --- | --- | --- | --- | --- | --- | --- | --- | --- | --- |
| Winter wheat | 0.33 | 0.24 | 0.42 | 1.14 | 1.42 | 0.73 |  |  |  | 0.85 | 0.92 | 0.54 |
| Summer maize |  |  |  |  |  | 0.65 | 0.84 | 0.94 | 1.34 |  |  |  |
| cotton |  |  |  | 0.38 | 0.38 | 0.53 | 1.00 | 1.07 | 1.28 | 0.78 |  |  |
| Sweet potato |  |  |  | 1.05 | 1.05 | 0.70 | 0.70 | 0.70 | 0.70 | 0.70 |  |  |
| peanuts |  |  |  | 0.95 | 0.95 | 0.95 | 0.55 | 0.55 |  |  |  |  |
