# Supplementary material for: Recharge and Groundwater Use in the North China Plain for Six Irrigated Crops for an Eleven Year Period
Source: PLoS One. 2015 Jan 27;10(1):e0115269. doi: 10.1371/journal.pone.0115269 (PMC4308074; doi:10.1371/journal.pone.0115269)
Supplement: S2 Table — (DOC) [file pone.0115269.s003.doc]

**Supporting Information**

**Table S2 Net water use (recharge minus irrigation) of each crop in each crop rotation of each year’s growing season from 2003 to 2013 in the North China Plain (mm).**

|  | SpCSpWS^*^ | | RCPWS^*^ | | PWS^*^ | | WS^*^ | | Cont C^*^ | |
| --- | --- | --- | --- | --- | --- | --- | --- | --- | --- | --- |
|  | crop | NWU^∮^ | crop | NWU | crop | NWU | crop | NWU | crop | NWU |
| 2003 | Sweet potato | -47^‡^ | Ryegrass | 4 | peanuts | -81 | Winter wheat | -96 | cotton | -64 |
|  |  |  | Cotton | -116 |  |  | Summer maize | -33 |  |  |
| 2004 | cotton | 116 | Peanuts | 184 | Winter wheat | -225 | Winter wheat | -225 | cotton | 134 |
|  |  |  |  |  | Summer maize | 125 | Summer maize | -6 |  |  |
| 2005 | Sweet potato | -125 | Winter wheat | -159 | peanuts | -90 | Winter wheat | -225 | cotton | -174 |
|  |  |  | Summer maize | -105 |  |  | Summer maize | -105 |  |  |
| 2006 | Winter wheat | -225 | Ryegrass | -150 | Winter wheat | -225 | Winter wheat | -225 | cotton | -64 |
|  | Summer maize | -105 | Cotton | -104 | Summer maize | -99 | Summer maize | -49 |  |  |
| 2007 | Sweet potato | -150 | Peanuts | -17 | peanuts | -143 | Winter wheat | -225 | cotton | -87 |
|  |  |  |  |  |  |  | Summer maize | -105 |  |  |
| 2008 | cotton | 20 | Winter wheat | -84 | Winter wheat | -162 | Winter wheat | -62 | cotton | 103 |
|  |  |  | Summer maize | -105 | Summer maize | -55 | Summer maize | -105 |  |  |
| 2009 | Sweet potato | -14 | Ryegrass | -150 | peanuts | -39 | Winter wheat | -225 | cotton | 21 |
|  |  |  | cotton | 16 |  |  | Summer maize | -95 |  |  |
| 2010 | Winter wheat | -225 | peanuts | -150 | Winter wheat | -225 | Winter wheat | -225 | cotton | -144 |
|  | Summer maize | -105 |  |  | Summer maize | -105 | Summer maize | -84 |  |  |
| 2011 | Sweet potato | -71 | Winter wheat | -225 | peanuts | -137 | Winter wheat | -225 | cotton | -160 |
|  |  |  | Summer maize | -105 |  |  | Summer maize | -74 |  |  |
| 2012 | cotton | 18 | Ryegrass | -150 | Winter wheat | -225 | Winter wheat | -225 | cotton | 69 |
|  |  |  | cotton | 112 | Summer maize | 48 | Summer maize | 54 |  |  |
| 2013 | Sweet potato | -38 | peanuts | 59 | peanuts | 89 | Winter wheat | -225 | cotton | 54 |
|  |  |  |  |  |  |  | Summer maize | -63 |  |  |

^∮^NWU denotes net water use of each crop from planting to harvesting (mm).

^‡^ Negative indicates the net groundwater depletion.

^*^ SpCSpWS rotation: sweet potato→ cotton→ sweet potato→ winter wheat–summer maize; WS rotation: winter wheat–summer maize; PWS rotation: peanuts→winter wheat–summer maize; RCPWS rotation: rye–cotton→ peanuts→ winter wheat–summer maize; Cont C: continuous cotton cropping system.
